# *GNA13* promotes brain metastasis of non-small cell lung cancer and EMT through the WNT/β catenin signaling pathway

**DOI:** 10.3389/fcell.2025.1652200

**Published:** 2025-09-26

**Authors:** Jia-Qi Wu, Han-Peng Zou, Ruo-Yue Fan, Jing Cai, Ping Wei, Ming-Fang He, Jin Xiang

**Affiliations:** ^1^ College of Biotechnology and Pharmaceutical Engineering, Nanjing Tech University, Nanjing, China; ^2^ Department of Science and Technology, The Affiliated Cancer Hospital of Nanjing Medical University and Jiangsu Cancer Hospital and Jiangsu Institute of Cancer Research, Nanjing, China

**Keywords:** NSCLC, brain metastasis, GNA13, zebrafish, Wnt/β catenin

## Abstract

**Background:**

Brain metastasis (BM) remains a major challenge in non-small cell lung cancer (NSCLC) treatment, with poorly understood mechanisms.

**Methods:**

A zebrafish xenograft model was established using the H1299 cell line to study NSCLC BM. RNA-Seq identified differentially expressed genes (DEGs) in metastasized to brain vs. non-metastasized cells. Clinical relevance of DEGs was validated using The Cancer Genome Atlas (TCGA) and Kaplan-Meier survival analysis. The anti-proliferation, migration, and invasion effects of *GNA13* were also detected using CCK8 assay, scratch wound healing assay, and transwell assay *in vitro*. The *in vivo* effects of *GNA13* in proliferation and migration were further examined in zebrafish embryos. Additionally, real-time quantitative PCR (RT-qPCR) and Western Blot were performed to validate and explore the underlying molecular mechanisms.

**Results:**

Through comprehensive RNA-Seq analysis of zebrafish xenograft model, we identified 177 DEGs significantly associated with NSCLC BM. KEGG and GO enrichment identified *GNA13* as a key mediator in NSCLC BM. Clinical correlation analysis confirmed that *GNA13* expression was associated with NSCLC BM and poor prognosis in lung cancer patients. Functional validation revealed that *GNA13* knockdown reduced H1299 cell viability, migration, and invasion, whereas overexpression in A549 cells increased viability migration, and invasion *in vitro*. These *in vitro* findings were further validated *in vivo*, where *GNA13* overexpression promoted tumor proliferation and metastatic potential. *GNA13* was shown to activate the Wnt/β-catenin signaling pathway and induce epithelial-mesenchymal transition (EMT), thereby enhancing the metastatic potential of lung cancer cells.

**Conclusion:**

This study identifies *GNA13* as a key gene of lung cancer BM. *GNA13* promotes EMT and enhances the proliferation and metastatic capacity of lung cancer cells by activating the Wnt/β-catenin signaling pathway. These findings suggest that *GNA13* may serve as a potential therapeutic target for preventing or treating BM in NSCLC.

## 1 Introduction

Lung cancer is the most frequently diagnosed cancer and the leading cause of cancer-related deaths worldwide, including in China (Zheng et al., 2024; Bray et al., 2024). Non-small cell lung cancer (NSCLC) was the predominant pathological phenotype of lung cancer, accounting for about 80% of total lung cancer cases (Ganti et al., 2021). Most cancer-related deaths are caused by metastasis. The brain is the most common site of NSCLC metastasis, reported in 25%–40% of cases (Chakrabarty et al., 2023; Yousefi et al., 2017). NSCLC patients with brain metastasis (BM) have a poor prognosis, with a median 5-year survival rate of less than 5%, which greatly affects patient survival and life quality (Sampson et al., 2020).

The current standard treatment for NSCLC BM patients involves surgery, radiotherapy, chemotherapy, targeted therapy, and anti-angiogenic therapy (Duan et al., 2023). Surgical resection is a localized therapeutic intervention used for selected patients as many were excluded from surgery due to bad performance status (Vogelbaum et al., 2022). Whole-brain radiotherapy (WBRT) is the standard treatment for symptom relief. While it may slightly improve survival, its toxic effects could lead to delayed neurological injury (Zhang et al., 2023). Stereotactic radiosurgery (SRS) is recommended only for patients with a limited number or volume of BM (Hosoya et al., 2024).

Drug resistance is a significant challenge to the clinical treatment of LCBM patients (Nikolaou et al., 2018). Although molecular mutations can be treated with targeted drugs, the following three limitations remain: (i) Only 10%–30% of NSCLC patients can benefit from targeted therapy (Goldberg et al., 2015); (ii) Targeted drugs were initially developed for the treatment of lung cancer, but their design did not take into account the permeability of these drugs to the blood-brain barrier (BBB). As a result, some targeted drugs have limited effectiveness in treating BM. (iii) NSCLC BM may exhibit significant genetic alterations compared with lung cancer cells from primary tumors, thus limiting the therapeutic effect of targeted treatments. In 86 NSCLC BM patients, whole exome sequencing revealed that 53% of BM expressed genetic alterations not found in matched primary tumor samples (Brastianos et al., 2015). EGFR, MET, and TP53 mutations were significantly expressed in primary and systemic metastases of NSCLC cells but were rarely found in NSCLC BM cells (7.7%) (Pellerino et al., 2021). The signaling pathways involved in cells with NSCLC BM may differ from those of primary tumors and other metastases. In addition, patient-to-patient and intratumoural heterogeneity can lead to variable efficacy of targeted therapies. These challenges highlight the need for improved treatment strategies and the identification of biomarkers for early detection of NSCLC BM. A deeper understanding of the pathogenesis of NSCLC BM is essential for developing new therapeutic agents and improving patient outcomes.

In previous studies, we have successfully established a zebrafish NSCLC BM model (Fan et al., 2021). In this study, we utilize the zebrafish NSCLC BM model to further investigate the mechanism of BM development. Through transcriptome sequencing and clinical relevance analysis, we have identified *GNA13* as a potential key gene involved in mediating BM. The G-protein subunit Alpha 13 (GNA13) was a member of the G Protein-coupled Receptors (GPCR) family, which is involved in the development and progression of various cancers. Previous studies have found that the GNA13 is upregulated in multiple cancers, such as gastric, colon, and hepatocellular carcinoma (Yi et al., 2023; Xu et al., 2016; Pan et al., 2022). In gastric cancer, high expression of GNA13 is associated with the activation of epithelial-mesenchymal transition (EMT) pathways, which drive the proliferation of gastric cancer cells (Zhang et al., 2016). Furthermore, GNA13 overexpression has been demonstrated to enhance the invasive and metastatic potential of colorectal cancer cells by modulating EMT-related processes (Pan et al., 2022). Na et al. reported that GNA13 could promote lung squamous cell carcinoma cell proliferation and migration by regulating the PI3K/AKT signaling pathway (Na et al., 2022). However, its role in NSCLC BM remains underexplored. Thus, this study aims to explore the role of *GNA13* in NSCLC BM, providing a new mechanism and new therapeutic target for NSCLC treatment.

## 2 Materials and methods

### 2.1 Regents

Tricaine, 1-phenyl-2-thio-urea (PTU), protease, and DMSO were purchased from Sigma-Aldrich (St. Louris, MO, United States). CM-DiI was purchased from Life Technologies (CA, United States). Fetal bovine serum (FBS) and Roswell Park Memorial Institute basal medium 1640 (RPMI 1640) were purchased from Basal Media Technologies (Shanghai, China).

### 2.2 Cell culture

NSCLC cell lines A549 and H1299 were obtained from the American Type Culture Collection (ATCC). A549 and H1299 cells were cultured in RPMI 1640 supplemented with 10% FBS at 37°C with 5% CO_2_.

### 2.3 Zebrafish and maintenance

The transgenic zebrafish [Tg (*fli-1*: EGFP)] which expresses enhanced green fluorescent protein (EGFP) in endothelial cells was obtained from the Model Animal Research Centre of Nanjing University. Zebrafish were handled as we previously reported (Fan et al., 2021). Briefly, adult zebrafish around 5–10 months of age with a 1:2 male-to-female ratio were used for mating. The zebrafish embryo age was expressed as hours post fertilization (hpf) or days post injection (dpi). The zebrafish studies were approved by the Institutional Animal Care and Use Committee (IACUC) at Nanjing Tech University.

### 2.4 Zebrafish xenografted and quantification

Zebrafish embryos at 24 hpf were dechorionated using protease (1 mg/mL, Sigma-Aldrich) and then placed in a PTU-containing E3 medium to prevent pigmentation. A549 and H1299 cells were labeled with CM-DiI according to the manufacturer’s instructions. Zebrafish embryos at 48 h post-fertilization (hpf) were anesthetized with tricaine. Approximately 100 cells of A549 or H1299 were injected into the perivitelline space (PVS) using a microinjector (IM-31, Narishige, Japan) for metastasis modeling. Additionally, 200-300 cells of A549 or H1299 were injected into the yolk sac to quantify cell proliferation. The injected zebrafish embryos were kept at room temperature for 1 h and then placed in an incubator at 32 °C until the end of the experiment. At 1 dpi, the successfully injected zebrafish embryos were collected under the microscope for subsequent experiments, as we have previously reported (Fan et al., 2021). For the zebrafish xenograft experiments, each experimental condition included more than 30 embryos and was independently repeated three times. Tumor cell proliferation in zebrafish embryos was calculated according to Formula 1 [Disp-formula e3]:

$$Fold\;changes=\frac{cell\;numbers\;of\;embryos\;at\;4\;dpi\;}{cell\;numbers\;of\;embryos\;at\;1\;dpi}$$ (1)

Formula 1, [Disp-formula e3] Tumor cell proliferation of NSCLC cells.

BM and tail metastasis (TM) cells in zebrafish embryos were calculated according to Formulas 2, 3, respectively.

$$Fold\;changes=\frac{the\;number\;of\;brain\;metastasis\;cells\;at\;4\;dpi}{the\;number\;of\;brain\;metastasis\;cells\;at\;1\;dpi}$$ (2)

Formula 2, [Disp-formula e5] Tumor cell BM of NSCLC cells

$$Fold\;changes=\frac{the\;number\;of\;tail\;metastasis\;cells\;at\;4\;dpi}{the\;number\;of\;tail\;metastasis\;cells\;at\;1\;dpi}$$ (3)

Formula 3, [Disp-formula e5] Tumor cell TM of NSCLC cells.

### 2.5 Collection and preparation of zebrafish tissues for sequencing

Brain tissue, tail tissue, and injection site tissue of 265 the H1299 cells xenografted zebrafish embryos were isolated and collected (Fan et al., 2021). The collected zebrafish tissues were subjected to RNA extraction, purification, and library construction, followed by paired-end sequencing using Next-generation sequencing, based on the Illumina sequencing platform (Hiseq X ten platform).

### 2.6 Bioinformatic analysis of transcriptome sequencing

Upon receiving the sequencing data, we conduct a quality assessment. Raw sequencing data achieved an average Q30 score >85% with 20 million reads per sample (30 Gb total depth). Read alignment to the reference genome using HISAT2 with default parameters, achieving an average alignment rate of 93.97%. We employed DESeq2 for statistical analysis, using default parameters for read count normalization and dispersion estimation. Genes with adjusted p-values <0.05 (Benjamini–Hochberg correction) and |log2FC| > 2 were considered differentially expressed. Then we have to normalize the Read Count of the genes by HTSeq software. The gene expression differential fold can be obtained by DESeq software, and the genes with adjusted p-values <0.05 and the expression differential fold |log2 FoldChange| > 2 are considered to be differentially expressed genes (DEGs). Differential gene volcano maps were drawn by Sangerbox 3.0 (http://vip.sangerbox.com/home.html). Then we used the R language Pheatmap package to perform cluster analysis on the screened differential genes, and the hierarchical clustering results of differential genes could be obtained. Gene ontology GO enrichment analysis using top GO software annotates differential genes to GO terms, counts the number of differential genes in each term, and calculates the P-value by the hypergeometric distribution method to filter out GO terms significantly enriched in differential genes with a P-value <0.05, thus identifying the major biological functions in which the differential genes are involved. Similarly, KEGG (http://www.kegg.jp/) enrichment analysis of differential genes was performed using DAVID (https://david.ncifcrf.gov/), and KEGG Pathway with significant enrichment of differential genes (P value <0.05 and |log2FC|> 2)was screened according to the P-value to identify the metabolic pathways and signaling pathways in which the differential genes are mainly involved. Differential genes significantly enriched for functions and pathways were mapped using ShinyGO v0.75 (http://bioinformatics.sdstate.edu/go/).

After GO enrichment and KEGG enrichment analysis, the enriched differential genes were subjected to clinical relevance studies to assess their association with the clinic. Clinical data on lung cancer patients were downloaded through the TCGA (https://www.cancer.gov/) and UCSC Xena databases (TCGA Lung Adenocarcinoma (LUAD) cohort, https://xenabrowser.net/heatmap/) and graphically analyzed using GraphPad Prism 9.0. DEGs were evaluated further with Kaplan–Meier Plotter database (https://kmplot.com/analysis/index.php?p=service&cancer=lung).

### 2.7 Quantitative real-time PCR (qRT-PCR)

Trizol reagent (Yifeixue, Nanjing, China) was used for the NSCLC cells to extract total RNA. Then total RNA was reverse transcribed to cDNA using a 1st Strand cDNA Synthesis kit (Yifeixue, Nanjing, China). The real-time PCR assay was performed using the 7900 HT system (ABI, United States) and SYBR green PCR kit (Yifeixue, Nanjing, China) to detect the expression levels of genes. The *GAPDH* gene was used as an internal standard. The thermal cycle procedure was as follows: 95 °C for 30 s; then 40 cycles of 95 °C for 10 s and 60 °C for 30 s. The relative quantification of PCR results was determined by -ΔΔCT. The sequences of primers are listed in Supplementary Table S1. The experiments were performed with three biological replicates (independent samples).

### 2.8 Cell transfection

A549 cells overexpression *GNA13* (A549-pcDNA-*GNA13* group) and the matched control cells were established using Lipo fectamine2000 carrying the pcDNA plasmid (Gene Pharma, shanghai, China). H1299 cells were transfected with siRNA (Gene Pharma, shanghai, China) to knock down *GNA13* (H1299-si*GNA13* group) using Lipo fectamine 2000. The three *GNA13* siRNA sequences used were as follows: siRNA#1 Forward primer 5′→3′GGCGUCGAGAAUUUCAACUTT and Reverse primer 5′→3′AGUUGAAAUUCUCGACGCCTT; siRNA#2 Forward primer 5′→3′CCUGCUAUAAGAGCAUUAUTT and Reverse primer 5′→3′AUAAUGCUCUUAUAGCAGGTT; siRNA#3 Forward primer 5′→3′GGUGGUCAGAGAUCAGAAATT and Reverse primer 5′→3′UUUCUGAUCUCUGACCACCTT. Cells were harvested after 24 or 48 h of transfection, and then gene knockdown and overexpression efficiencies were quantified by qRT-PCR. “H1299-control” represents the untransfected wild-type cell control, while “H1299-NC” denotes the negative control group transfected with scrambled siRNA. Similarly, “A549-control” refers to the wild-type cells without overexpression, and “A549-NC” indicates the negative control group overexpressed with scrambled pcDNA vector.

### 2.9 Cell viability assay

Transfected NSCLC cells were seeded in 96-well plates at 4 × 10^3^ cells/well. Cell viability was determined by the CCK-8 assay (Vazyme, Nanjing, China) following the manufacturer’s instructions. The absorbance at a wavelength of 450 nm was then measured using a microplate reader (BioTek, Winooski, VT, United States) after 1 and 2 days.

### 2.10 Cell wound healing

For the NSCLC cells wound healing assay, 2.5 × 10^5^ cells of A549 or H1299 cell line were seeded in 6-well plates, and vertical scratches were made using a 1 mL pipet tip after overnight incubation. Cells were then cultured in a medium with 2% FBS for 24 h. Cell migration rate was measured using Formulas 4, 5.

$$average\;scratch\;width=\frac{scrath\;area}{scrath\;length}$$ (4)

Formula 4, [Disp-formula e5] Mean value of scratch width

$$cell\;mobility\%=\frac{0\;h\;scratch\;width-24\;h\;scratch\;width}{0\;h\;scratch\;width}\times 100\%$$ (5)

Formula 5 Cell mobility.

### 2.11 Cell invasion

After 24 h of serum-free culture, 5 × 10^4^ cells were seeded in the upper chamber (pre-coated with Matrigel) with 200 μL medium with 2% FBS. The lower chamber was filled with 500 μL medium with 10% FBS. The invasion chamber was incubated for 24 h, then the upper chamber was washed, methanol fixed, stained with 0.5% crystal violet solution and photographed.

### 2.12 Western blot

The total protein from H1299-control, H1299-NC, H1299-siRNA, A549-control, A549-NC, and A549-pcDNA was extracted using RIPA lysis buffer (Beyotime, Shanghai, China) with 1% PMSF (Beyotime, Shanghai, China). The concentration was measured using the BCA kit (Beyotime, Shanghai, China) according to the manufacturer’s instructions. According to the Western blotting protocol described, the level of protein investigated were evaluated with the following antibodies: N-cadherin Polyclonal antibody (22018-1-AP, 1: 2000), Snail-1 Polyclonal antibody (13099-1-AP, 1: 1000), Snail-2 Polyclonal antibody (12129-1-AP, 1: 1000), and ZEB-2 Polyclonal antibody (14026-1-AP, 1: 1000) were purchased from Proteintech (wuhan, China). ZEB-1 Rabbit mAb (3396, 1: 1000), GSK3β Rabbit mAb (9315, 1: 1000), and phos-GSK3β (Ser9) Rabbit mAb (9322, 1: 1000) were purchased from CST (Boston, United States). active β-catenin (05-665, 1: 1000) was purchased from Sigma-Aldrich (St. Louris, MO, United States). Anti-β-catenin (sc-7963, 1: 1000) was purchased from Santz Cruz (CA, United States). Anti-β-actin (ab8226, 1: 1000), Goat Anti-Rabbit IgG H&L (HRP) (ab6721, 1: 10000), and Rabbit Anti-Mouse IgG H&L (HRP) (ab6728, 1: 10000) were purchased Abcam (Cambridge, United Kingdom).

### 2.13 Imaging

All images were obtained in a Zeiss fluorescence microscope (Axio vert A1, Zeiss, Germany) or an Olympus fluorescence microscope (IX71, Olympus, Tokyo, Japan). We used ImageJ to analyze and quantify the images.

### 2.14 Data statistics

All statistical analyses were expressed as mean ± SEM using GraphPad Prism 9.0. Data were analyzed using either unpaired t-tests or one-way/two-way ANOVA, as appropriate for each experimental design, with Tukey’s test employed for multiple comparisons when ANOVA was applied. Variance homogeneity was confirmed through Brown-Forsythe testing (all p > 0.05), while normality was verified for each experimental group using Shapiro-Wilk tests (all p > 0.05). Significance was considered when P values were lower than 0.05. All experiments were done in triplicates.

## 3 Results

### 3.1 Identification of key genes in injecting site to BM and TM in zebrafish NSCLC BM model

**FIGURE 1:**
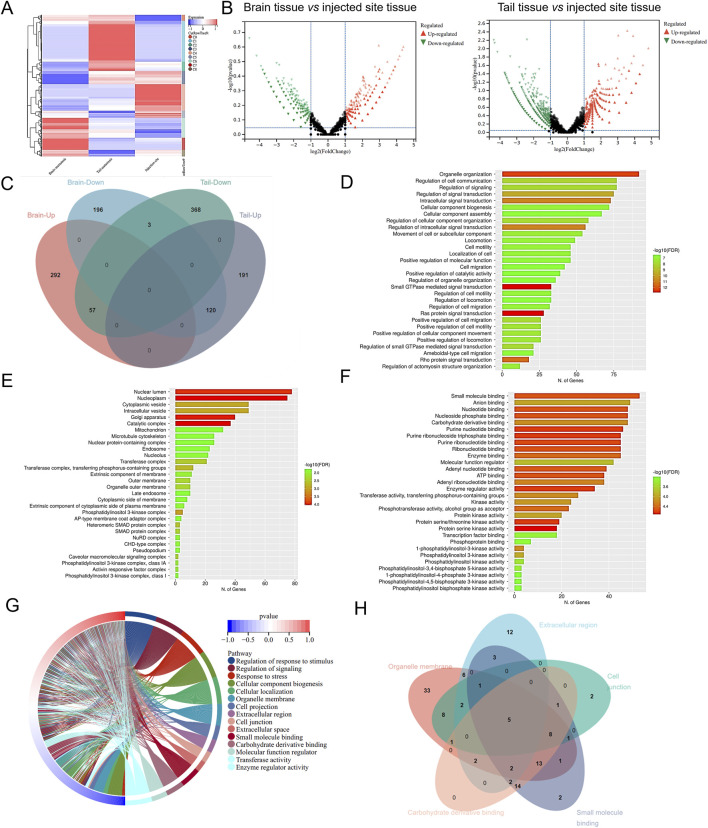
Identification of key genes in injecting sites to brain or tail metastasis in zebrafish-NSCLC-BM model. **(A)** Cluster analysis of gene expression levels in brain metastasis, TM, and injection site. **(B)** Volcanic map of DEGs in brain or tail metastasis compared with *in situ* injection. **(C)** Venn diagrams of differential genes in brain and tail metastasis. The top 30 items with the most significant enrichment of the GO Biological Process **(D)**, GO Cellular Component **(E)**, and GO Molecular Function **(F)**. **(G)** GO enrichment analysis circle diagram. **(H)** Venn diagram showing the DEGs in GO enrichment analysis.

To explore the underlying mechanism in the BM of NSCLC cancer, we employed RNA-seq using brain tissue, tail tissue, and the rest tissues of 265 xenografted zebrafish. As shown in Figure 1A, the data FPKM normalized by the R language was plotted as a cluster analysis of gene expression in the BM, TM, and injection site group. The gene expression of the injected site group was used as a control, and the gene expression of the BM tissues and TM tissues were processed for DEGs analysis. We used the absolute values of log2FoldChange >2 as the screening criteria to identify differential genes, which were then plotted as volcanoes (Figure 1B). Compared to gene expression at the injection site tissue, BM tissue exhibited 668 DEGs, with 349 genes upregulated and 319 genes downregulated. TM tissue showed 739 DEGs, comprising 428 upregulated genes and 311 downregulated genes. Next, we plotted the Venn map to compare the up- and downregulated DEGs in brain and TM tissues (Figure 1C). In the brain and tail metastasis tissues, we identified 177 DEGs with opposite trends. Among these, 57 DEGs were upregulated in BM tissue but downregulated in TM tissue, and 120 DEGs were downregulated in BM but upregulated in TM tissue.

**FIGURE 2:**
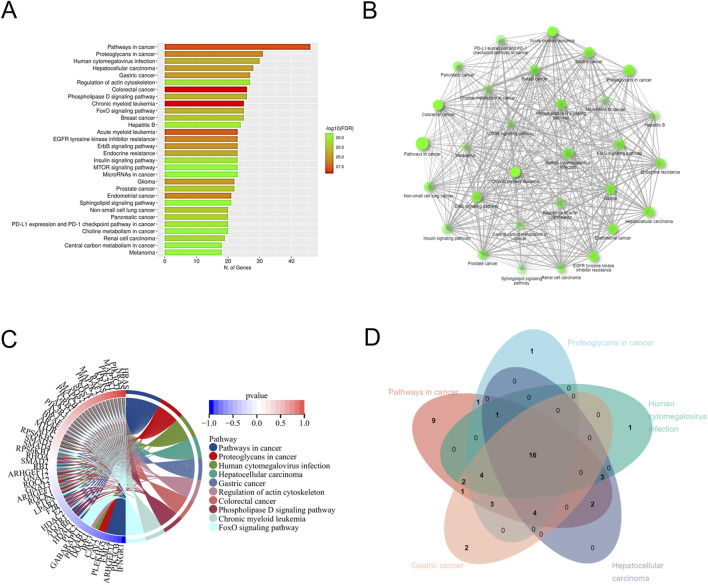
KEGG enrichment analysis of DEGs. **(A)** The top 30 pathways with the highest significance in KEGG concentration are displayed. **(B)** KEGG enrichment network diagram. **(C)** KEGG enrichment analysis circle diagram. The left semicircle represents DEGs involved in KEGG enrichment, while the outer semicircle represents DEGs involved in the KEGG pathway. The right semicircle shows 10 KEGG pathways linked by lines to genes in the left semicircle. **(D)** Venn diagram shows DEGs in the KEGG enrichment analysis.

The 177 DEGs were analyzed by performing GO and KEGG enrichment. GO analysis classified DEGs into three main categories, biological processes (BP) (Figure 1D), cellular component (CC) (Figure 1E), and molecular function (MF) (Figure 1F). The top 15 items in the BP, CC, and MF were plotted as an enrichment analysis circle diagram (Figure 1G). The results show that the parts of the DEGs are involved in multiple GO items. The first five GO items were analyzed using a Venn diagram (Figure 1H), and the five DEGs with strong and significant involvement were obtained, including *RHOA*, *GNA13*, *MAPK1*, *MAPK2K2*, and *BMPR2*. KEGG analysis revealed that 177 DEGs are mainly associated with cancer-related pathways (Figure 2A). The results of the KEGG enrichment analysis were plotted as a network diagram showing the correlation between KEGG-enriched pathways (Figure 2B). The top ten pathways with the highest correlation of KEGG enrichment were plotted into a KEGG enrichment analysis circle diagram (Figure 2C), which showed that some DEGs were involved in multiple KEGG pathways. The top five pathways were selected from the KEGG circle diagram and plotted into a Venn diagram (Figure 2D), the top 16 DEGs with the highest involvement and significance in KEGG-enriched pathways were *PIK3CB*, *RHOA*, *MAPK1*, *SOS1*, *SOS2*, *PIK3R1*, *GNA13*, *PIK3CA*, *RAF1*, *KRAS*, *PIK3R3*, *MAP2K1*, *MAP2K2*, *PIK3CD*, *NRAS*, and *HRAS*. By integrating these GO and KEGG-derived gene sets and eliminating redundancies, we ultimately identified 17 DEGs (*PIK3CB*, *RHOA*, *MAPK1*, *SOS1*, *SOS2*, *PIK3R1*, *GNA13*, *PIK3CA*, *RAF1*, *KRAS*, *PIK3R3*, *MAP2K1*, *BMPR2*, *MAP2K2*, *PIK3CD*, *NRAS*, and *HRAS*) associated with lung cancer. They may play an important role in the progression of brain metastasis in lung cancer.

**FIGURE 3:**
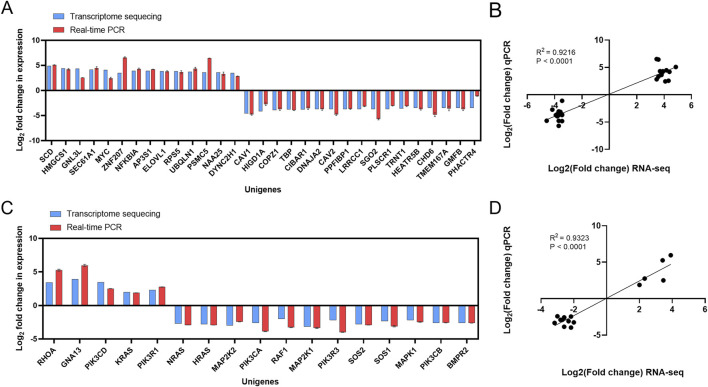
Validation of transcriptome sequencing results. **(A)** Comparison of RNA-seq results and qPCR results. **(B)** Correlation analysis between RNA-seq results and qPCR results. **(C)** Comparison of RNA-seq results of key DEGs with qPCR results. **(D)** Correlation analysis between RNA-seq results of key DEGs and qPCR results.

The 31 DEGs were randomly selected from 177 DEGs and then validated by RT-qPCR. As shown in Figures 3A,B, the log2 fold change of gene expression in RT-qPCR experiments was consistent with the results in RNA-seq regarding up- and downregulation trends. Then, the 17 key DEGs that had been selected by GO and KEGG analysis were also verified by RT-qPCR. The correlation analysis demonstrates excellent concordance between RT-qPCR and RNA-seq results, with highly significant Pearson correlation coefficients of *R* ^2^ = 0.9216 (P < 0.0001) and *R* ^2^ = 0.9323 (P < 0.0001) for the DEGs (Figures 3C,D).

### 3.2 GNA13 is highly expressed in metastasis NSCLC and associated with poor prognosis

**FIGURE 4:**
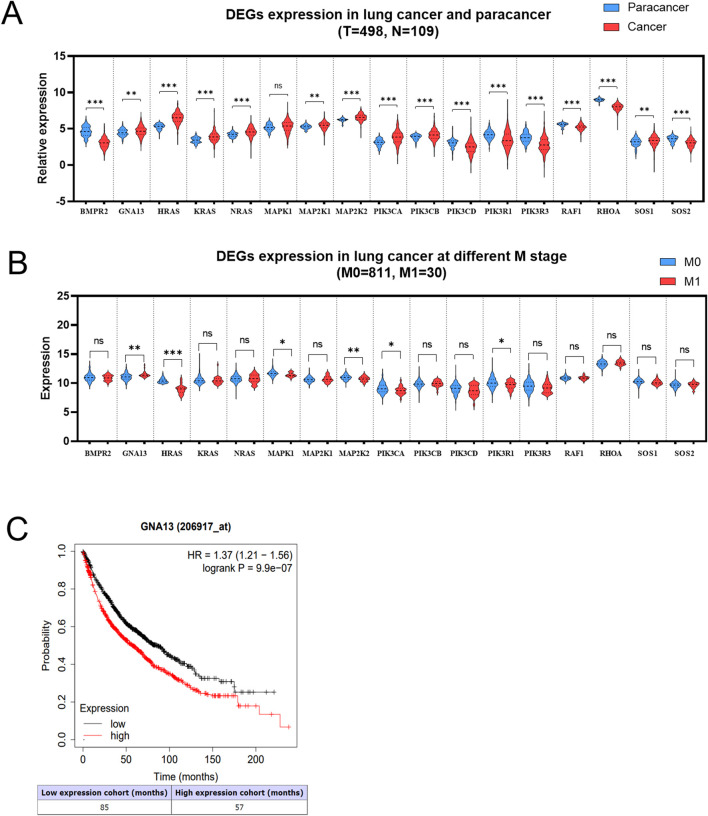
*GNA13* is highly expressed in metastasis NSCLC and associated with poor prognosis. **(A)** Relative expression of DEGs in paracancer and tumor tissues of lung cancer. The data was obtained from the TCGA database. **(B)** Expression of DEGs in lung cancer patients at stage M. The data was obtained from the UCSC Xena database. **(C)** The Kaplan-Meier analyses of survival of lung cancer patients with high (red) and low (black) expression levels of *GNA13*. The cutoff for *GNA13* expression levels was determined using the median expression value. Data was analyzed by an unpaired t-test. (ns) indicated statistical insignificance, (*) P < 0.05, (**) P < 0.01, and (***) P < 0.001.

The pan-cancer analysis was performed to determine whether 17 DEGs were expressed in NSCLC tissues. The results suggested that 7 DEGs expressions were downregulated in lung cancers compared with normal tissues, including *BMPR2*, *PIK3CD*, *PIK3R1*, *PIK3R3*, *RAF1*, *RHOA*, and *SOS2* (Figure 4A). It was also found that 9 DEGs (*GNA13*, *HRAS*, *KRAS*, *NRAS*, *MAP2K1*, *MAP2K2*, *PIK3CA*, *PIK3CB*, and *SOS1*) expressions were higher in lung cancers than in normal tissues (Figure 4A). However, the expression of *MAPK1* was no different in lung cancers relative to normal tissues, indicating that *MAPK1* may be irrelevant to lung cancer. Based on the UCSC Xena database (M0 = 811, M1 = 30), we examined the 16 DEGs expressions in lung cancers with/without metastasis. As shown in Figure 4B, 6 DEGs expressions are significantly different in lung cancers with or without metastasis. *GNA13* was upregulated (1.17-fold change, P = 0.001) in the M1 stage lung cancer than the M0 stage lung cancer. *HRAS* (0.92-fold, P < 0.001), *MAPK1* (0.87-fold, P = 0.01), *MAP2K2* (0.94-fold, P = 0.001), *PIK3RCA* (0.87-fold, P = 0.01), and *PIK3R1* (0.77-fold, P = 0.02) expression were low in the M1 stage lung cancer related to the M0 stage lung cancer. We combined the results of the pan-cancer database and the UCSC Xena database to compare the DEGs expression. *GNA13* expression was consistent between lung cancer vs*.* normal tissue and metastasis lung cancer vs. non-metastasis lung cancer (Table 1). Moreover, the Kaplan-Meier analysis suggested that *GNA13* high expression in lung cancer is associated with a short Median Survival Time (Figure 4C). Together, these results indicate that *GNA13* was identified as a key gene associated with lung cancer metastasis and poor prognosis.

**TABLE 1:** Clinical correlation analysis of DEGs.

Gene	Lung cancer vs. para cancer	M1 vs. M0
*GNA 13*	Upregulated	Upregulated
*HRAS*	Upregulated	Downregulated
*MAP2K2*	Upregulated	Downregulated
*PIK3RCA*	Upregulated	Downregulated
*PIK3R1*	Downregulated	Downregulated

### 3.3 GNA13 promotes NSCLC cell proliferation, migration, and invasion *in vitro*

**FIGURE 5:**
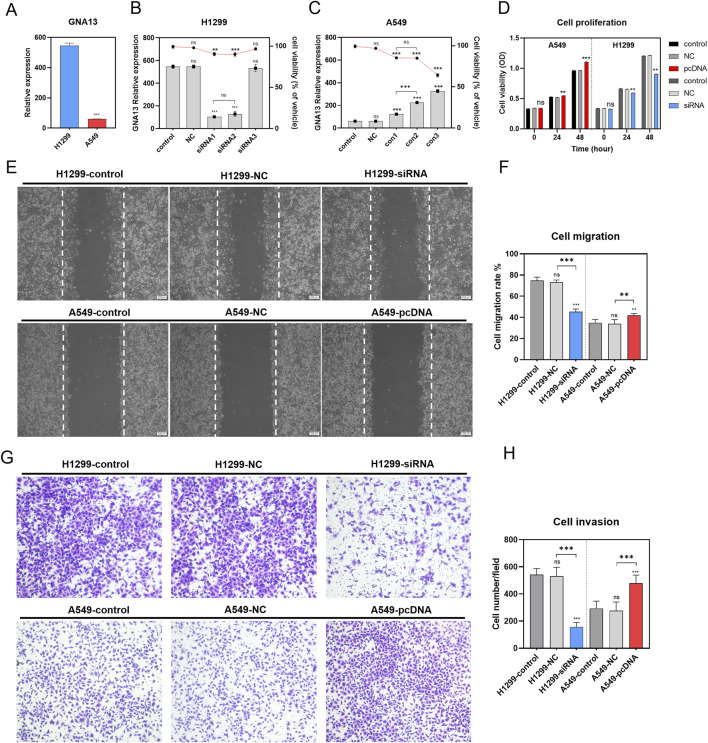
Effects of *GNA13* expression on lung cancer cell proliferation, migration, and invasion *in vitro*. **(A)** Relative expression of *GNA13* in lung cancer cell lines H1299 and A549. **(B)** Effects of idling group and three different *siRNA* on the viability of H1299 and the relative expression of *gna13* in H1299. The bar graph represents the relative expression of *GNA13* in H1299, and the line graph represents the cell viability of H1299. **(C)** Effects of the negative control group and three different concentrations of transfection reagents on the viability of A549 and the relative expression of *GNA13* in A549. The bar graph represents the relative expression of *GNA13* in A549, and the line graph represents the cell viability of A549. **(D)** Effect of *GNA13* expression on cell proliferation. **(E)** Images of cell scratches in cell migration experiments. The scratch wound was shown in the middle of the two white dashed lines in the figure. Scale bar: 200 μm. **(F)** Effect of *GNA13* expression on cell migration. **(G)** Crystal violet images of cells in Transwell invasion assay. **(H)** Effect of *GNA13* expression on cell invasion. Data was analyzed by one-way/two-way ANOVA followed by Tukey’s multiple comparisons test. (ns) indicated statistical insignificance, (*) P < 0.05, (**) P < 0.01, and (***) P < 0.001.

Next, we explored the important role of *GNA13* in NSCLC cells. We investigated whether knockdown or overexpression of *GNA13* expression using siRNA and Plasmid could affect cell proliferation, migration, and invasion, respectively. *GNA13* expression was evaluated in H1299and A549 cells. We observed that *GNA13* expression was significantly higher (P < 0.001) in H1299 cells than in A549 cells (Figure 5A). Hence, we used H1299 cells for knockdown and A549 cells for overexpression of *GNA13* in subsequent assays. The knockdown efficiency in H1299 cells and overexpression efficiency in A549 cells of *GNA13* were assessed by qRT-PCR. Si RNA-*GNA13* #1 showed the highest knockdown efficiency in H1299 cells and cell viability >91% (p = 0.001) (Figure 5B). pC-DNA-*GNA13*#3 (Lipo: plasmid = 4: 5) showed the highest overexpression efficiency in A549 cells but exhibited cytotoxicity (Figure 5C). pC-DNA-*GNA13*#1 (Lipid: Plasmid = 1:1) and pC-DNA-*GNA13*#2 (Lipid: Plasmid = 3:4) ensured 85% cell viability and pC-DNA#2 (Lipid: Plasmid = 3:4) showed a better effect on *GNA13* expression in A549 cells. Then, siRNA-*GNA13*#1 and pC-DNA-*GNA13*#2 were chosen for subsequent experiments.

We examined the effect of *GNA13* on cell viability using the CCK8 assay. As shown in Figure 5D, knockdown or overexpression of *GNA13* affected the viability of H1299 or A549 cells when transfected for 24 h. Knockdown of *GNA13* in H1299 cells resulted in a modest but statistically significant 10.1% reduction in cell viability (P = 0.005). Conversely, overexpression of *GNA13* in A549 cells led to a 3.6% increase in viability (P = 0.001). After 48 h of transfection, *GNA13* knockdown inhibited the proliferation of H1299 cells, while *GNA13* overexpression promoted the proliferation of A549 cells. Compared to the negative control group (H1299-NC group), *GNA13* knockdown reduced the migration of H1299 cells (H1299-si*GNA13*) in the wound healing assay (Figures 5E,F). In A549 cells, *GNA13* overexpression increased migration compared to the negative control group (A549-NC group). *GNA13* knockdown or overexpression showed similar results in the trans well assay. The *GNA13* knockdown group (H1299-si*GNA13*) exhibited fewer cell invasions, while the *GNA13* overexpression group (A549-pc-DNA-*GNA13*) showed more cell invasions than the negative control group (Figures 5G,H). Together, those results suggested that *GNA13* participated in the proliferation, migration, and invasion capabilities of NSCLC.

### 3.4 GNA13 promotes NSCLC cell proliferation, BM, and TM *in vivo*

**FIGURE 6:**
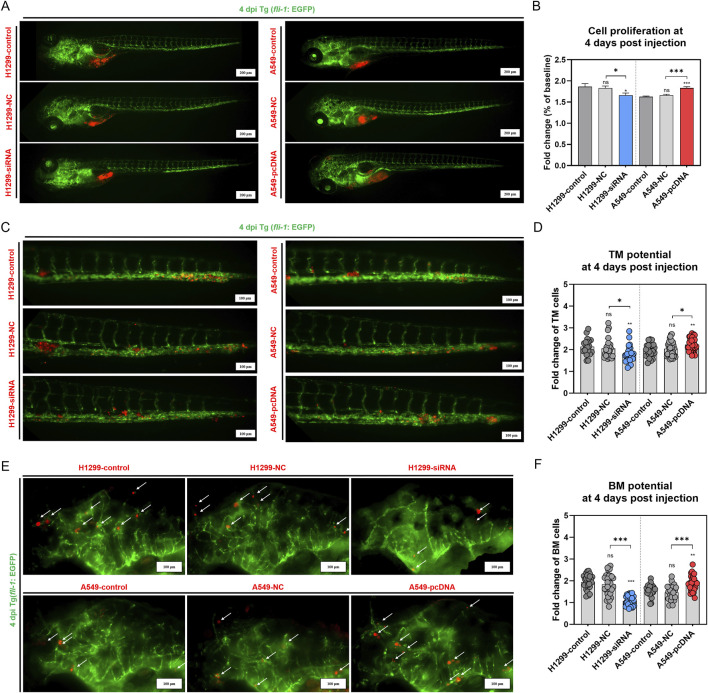
Effects of *GNA13* expression on the NSCLC cells proliferation, TM, and BM potential in zebrafish. **(A)** Images of lung cancer cell proliferation in zebrafish at 4 dpi. Scale bar: 200 μm. **(B)** Quantification of lung cancer cell proliferation in zebrafish at 4 dpi. **(C)** Images of lung cancer cell TM potential in zebrafish at 4 dpi. Scale bar: 100 μm. **(D)** Quantification of lung cancer cell TM potential in zebrafish at 4 dpi. **(E)** Images of lung cancer cell BM potential in zebrafish at 4 dpi. Scale bar: 200 μm. **(F)** Quantification of lung cancer cell BM potential in zebrafish at 4 dpi. Data was analyzed by one-way ANOVA followed by Tukey’s multiple comparisons test. (ns) indicated statistical insignificance, (*) P < 0.05, (**) P < 0.01, and (***) P < 0.001.

To further investigate the role of *GNA13* in tumor distance metastasis and proliferation *in vivo*, we use the zebrafish xenografted model. CM-DiI labeled H1299-control, H1299-NC, H1299-si*GNA13*, A549-control, A549-NC, and A549-pcDNA-*GNA13* cells were injected into the yolk sac of zebrafish embryos and monitored at day 1 and 4 postinjection. At 4 dpi, the H1299-NC group showed proliferation results similar to those of the H1299-control group. Compared with H1299-NC cells, the *GNA13* knockdown (H1299-si*GNA13*) reduced tumor cell numbers (Figures 6A,B). Consistently, the A549-NC and A549-control groups showed no difference in cell proliferation. Compared to the A549-NC cells, the overexpression of *GNA13* enhances the proliferation of A549-control cells (Figures 6A,B). Based on our previous study, we established the metastasis zebrafish xenograft model to investigate the effects of *GNA13* on metastasis. The CM-DiI labeled H1299-control, H1299-NC, H1299-si*GNA13*, A549-control, A549-NC, and A549-pcDNA-*GNA13* cells were injected into the perivitelline space of zebrafish embryos.

At 4 dpi, we quantified tumor cell numbers in the brains and tails of zebrafish embryos separately. Figures 6C,D showed a significant distant metastasis (brain and tail) in H1299 and A549 xenografted. The cell TM potential was 2.14 in the H1299-control group, 2.05 in the H1299-NC group, and 1.77 in the H1299-si*GNA13* group. The cell TM potential was 1.96 in the A549-control group, 2.04 in the A549-NC group, and 2.23 in the A549-pcDNA-*GNA13* group. The results indicated no significant difference in the cell TM potential between the H1299-control and the H1299-NC group (p = 0.75). However, the cell TM potential of the H1299-si*GNA13* cells was significantly lower than that of both the H1299-control cells (p = 0.008) and the H1299-NC cells (p = 0.04). In contrast, the A549-control cells showed no significant change in cell TM potential when compared to the A549-NC cells (p = 0.59). Notably, the A549-pcDNA-*GNA13* cells exhibited a significant enhancement in cell TM potential compared to the A549-control (p = 0.004) and A549-NC cells (p = 0.03). As shown in Figures 6E,F, the brain metastatic potential of the H1299-control group was 1.94, while the H1299-NC group had a potential of 1.85, and the H1299-si*GNA13* group exhibited a potential of 1.07. In A549 cells, the A549-control group had a potential of 1.56, the A549-NC group had 1.47, and the A549-pcDNA-*GNA13* group showed a potential of 1.87. Quantitative analysis showed a modest numerical difference in BM potential between H1299-control (1.94) and H1299-NC (1.85) cells. However, statistical analysis revealed this difference was not significant (p = 0.63), indicating comparable metastatic potential between these two groups. The brain metastatic potential of H1299-si*GNA13* cells was significantly decreased compared to H1299-control (p < 0.001) and H1299-NC cells (p < 0.001). The A549-control cells showed no significant change in BM potential compared to the A549-NC cells, whereas the A549-pcDNA-*GNA13* cells exhibited a significantly increased BM potential compared to both the control (p = 0.004) and NC cells (p < 0.001). The results indicated that knocking down *GNA13* expression reduced the proliferation, TM, and BM of NSCLC cells in zebrafish. Conversely, overexpressing *GNA13* increased the proliferation, TM, and BM of NSCLC cells in zebrafish.

### 3.5 GNA13 activates WNT/β-catenin signaling and EMT in NSCLC cell

**FIGURE 7:**
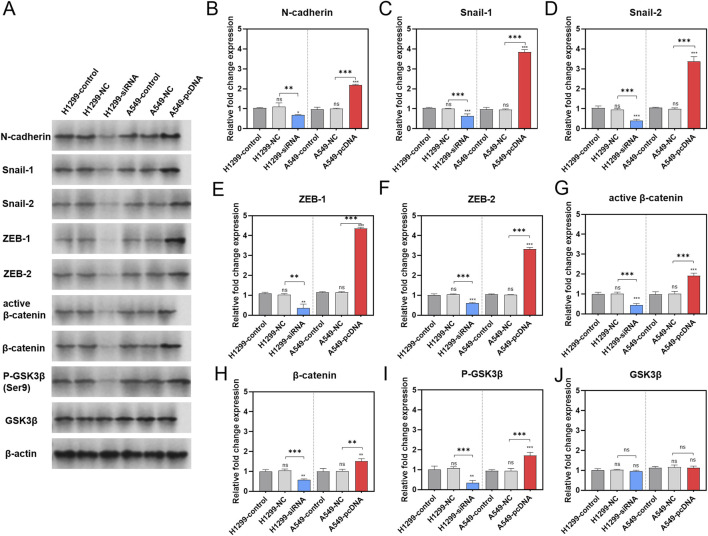
Effect of *GNA13* expression on the levels of proteins associated with EMT/WNT signaling pathway. **(A)** Representative images of each group are shown. Western blot analysis of N-cadherin **(B)**, Snail-1 **(C)**, Snail-2 **(D)**, ZEB-1 **(E)**, ZEB-2 **(F)**, active β-catenin **(G)**, β-catenin **(H)**, P-GSK3β **(I)**, and GSK3β **(J)** expression after *GNA13* knockdown or overexpression. Data was analyzed by one-way ANOVA followed by Tukey’s multiple comparisons test. (ns) indicated statistical insignificance, (*) P < 0.05, (**) P < 0.01, and (***) P < 0.001.

To identify the pathway underlying the effect of *GNA13* on proliferation and metastasis, EMT-related proteins (N-cadherin, snail-1, snail-2, zeb-1, and zeb-2) were examined by Western Blotting assay (Figure 7A). The results indicated that the expression levels of N-cadherin (Figure 7B), Snail-1 (Figure 7C), Snail-2 (Figure 7D), ZEB-1 (Figure 7E), and ZEB-2 (Figure 7F) proteins did not show significant differences between the H1299-NC cells and the H1299-control cells. However, the *GNA13* knockdown significantly decreased the expression levels of N-cadherin (p = 0.02), Snail-1 (p < 0.001), Snail-2 (p < 0.001), ZEB-1 (p = 0.001), and ZEB-2 (p < 0.001) proteins in H1299-si*GNA13* cells compared to H1299 control cells. In comparison to the control cells from A549, the A549-NC cells showed no significant change in the expression levels of N-cadherin (Figure 7B, p = 0.87), Snail-1 (Figure 7C, p = 0.99), Snail-2 (Figure 7D, p = 0.79), ZEB-1 (Figure 7E, p > 0.99), and ZEB-2 (Figure 7F, p > 0.99) proteins. The relative expression of N-cadherin (p < 0.001; p < 0.001), Snail-1 (p < 0.001; p < 0.001), Snail-2 (p < 0.001; p < 0.001), ZEB-1 (p < 0.001; p < 0.001), and ZEB-2 (p < 0.001; p < 0.001) proteins was significantly increased in *GNA13* overexpressing (A549-pcDNA-*GNA13*) cells compared to both A549-control cells and A549-NC cells. Those findings indicated that downregulated *GNA13* expression inhibited EMT progression, while upregulated *GNA13* expression promoted EMT progression in NSCLC cells.

The effects of *GNA13* expression on the levels of phos-GSK3β (Ser9), GSK3β, active β-catenin, and β-catenin proteins in the Wnt/β-catenin signaling pathway were also investigated (Figure 7A). The results showed that there were no significant changes in the expression of active β-catenin (Figure 7G, p = 0.83), β-catenin (Figure 7H, p = 0.83), phos-GSK3β (Ser9) (Figure 7I, p = 0.81), and GSK3β (Figure 7J, p = 0.77) proteins in H1299-NC cells compared to H1299-control cells. The expression levels of the above proteins in the A549-control group were similar to those in the A549-NC group. The levels of phos-GSK3β (Ser9) (p = 0.002), active β-catenin (p < 0.001), and β-catenin (p = 0.001) proteins were significantly downregulated in the H1299-si*GNA13* cells. However, the total protein expression of GSK3β remained unchanged. In contrast, in cells overexpressing *GNA13* (A549-pcDNA-*GNA13*), the expression of phos-GSK3β (Ser9) (p < 0.001), active β-catenin (p < 0.001), and β-catenin (p = 0.005) proteins was significantly upregulated, with no significant change observed in the total GSK3β (p > 0.99) protein expression. In summary, when the expression of *GNA13* was decreased in lung cancer cells, the level of Ser9 phosphorylation in GSK3β also decreased, while the total protein expression levels remained unchanged. Additionally, the protein levels of both β-catenin and active β-catenin were reduced, indicating that Wnt/β-catenin signaling was inhibited. Conversely, when *GNA13* was overexpressed in lung cancer cells, the expression level of GSK3β Ser9 phosphorylation increased, leading to elevated protein levels of both β-catenin and active β-catenin, which activated Wnt/β-catenin signaling.

## 4 Discussion

Due to lack of commercially brain-metastatic cell lines derived from human samples, we developed a zebrafish xenograft model for studying LCBM, which proved to be feasible and reliable (Fan et al., 2021). In this study, we collected lung cancer cells that had metastasized to either the brain or tail, as well as those that remained at the site of injection of the H1299 zebrafish LCBM xenografted. Using transcriptome sequencing, we aimed to identify key genes that mediate BM. While our initial DEG identification utilized FPKM normalization - chosen for its widespread adoption in zebrafish studies and ability to account for transcript length variations - we implemented a rigorous validation framework including DESeq2 analysis, *R* ^2^ platform verification, and clinical correlation with TPM-normalized TCGA data to ensure the robustness of our findings across different normalization methods. To obtain the key genes related to BM, we based our approach on the principle that brain and TM trends are mutually exclusive, given that a cell cannot be brain metastasized and TM at the same time. Genes with consistent trends between “BM vs. injection site” and “TM vs. injection site” can be considered differential genes that promote lung cancer cell migration away from the injection site and can be excluded. A total of 177 differential genes that were found to be identical in both the “BM vs. injection site” and “TM vs. injection site” groups, but exhibited inconsistent expression trends, were identified as key genes. These genes are believed to play a significant role in the ability of lung cancer cells to remain in either the brain or tail of zebrafish after metastasis. Through GO and KEGG enrichment analysis, we focused on 17 DEGs: *BMPR2*, *PIK3CB*, *RHOA*, *MAPK1*, *SOS1*, *SOS2*, *PIK3R3*, *GNA13*, *PIK3CA*, *RAF1*, *KRAS*, *PIK3R1*, *MAP2K1*, *MAP2K2*, *PIK3R1*, *NRAS*, and *HRAS*. *BMPR2* belongs to the brain metastasis P family and has been reported to be associated with the development of inflammation (Sánchez-Du et al., 2019), bladder cancer (Martínez et al., 2017), and breast cancer progression (Liu et al., 2021); The PI3K family plays a significant role in cancer progression, influencing both cell proliferation and resistance to apoptosis. Consequently, the development of targeted PI3K inhibitors has gained considerable attention in recent years (Yang et al., 2019; Fattahi et al., 2020); The Ras/Raf/MEK/ERK signaling pathway is closely linked to cancer. The four key genes of this pathway—*Ras*, *Raf*, *MEK*, and *ERK*—can trigger serious tumor diseases if they function abnormally (Rubio et al., 2023; Khojasteh et al., 2021; Lee et al., 2020; Chen et al., 2019; Asati et al., 2016); It has been shown that lung cancer cells activate Rho GTPase during trans-BBB, contributing to increased contractility of actinomyosin and resulting in the disruption of endothelial cell tight junctions in the BBB (Yousefi et al., 2017). Trans-BBB migration of lung cancer cells can be blocked by inhibiting the expression of *ROCK* of the Rho/ROCK pathway (Yousefi et al., 2017; Xu et al., 2020). GNA13 belongs to the largest family of cell-surface receptors known as G protein-coupled receptors (GPCRs). Studies have shown that the expression of *GNA13* increases as breast and prostate cancer cells become more invasive (Huang et al., 2019; Pan et al., 2020). Additionally, *GNA13* is recognized as a biomarker indicating a poor prognosis in gastric cancer (Yin et al., 2020).

To further investigate the key genes involved in the BM of lung cancer cells, we examined the clinical relevance of these 17 DEGs to lung cancer. Except for *MAPK1*, the remaining 16 DEGs were significantly differentially expressed in lung cancer tumor tissues (vs. para cancer tissues). Patients with brain metastases from lung cancer are classified as the M1 stage, indicating the presence of distant metastases beyond the lungs. In contrast, the M0 stage indicates that the patient has no distant metastases. The clinical relevance of the 16 DEGs to lung cancer metastasis was evaluated by analyzing whether their expression differed in tumor tissues of lung cancer patients with or without distant metastasis. The results of the analysis showed that *GNA13*, *HRAS*, *MAP2K2*, *PIK3CA*, and *PIK3R1* were correlated with distant metastasis of lung cancer. Among the five DEGs, *PIK3R1* (*) and *GNA13* (**) were consistently and significantly downregulated in the tumor tissues of lung cancer patients compared to normal tissues. Additionally, both showed significant downregulation in the tumor tissues of patients at stage M1 compared to those at stage M0. This indicates that *PIK3R1* and *GNA13* are relevant to lung cancer metastasis, with *GNA13* being more strongly associated with distant metastasis. Kaplan-Meier survival curves also showed a correlation between *GNA13* and poor prognosis in lung cancer. These consistent results across multiple analytical approaches strongly implicate *GNA13* as a potential key mediator in lung cancer BM.

However, the anti- BM effect of *GNA13* in lung cancer and its underlying mechanisms have not yet been elucidated. To confirm this hypothesis, loss-of-function and gain-of-function experiments were conducted for *GNA13* in NSCLC cell lines. In this study, we established transient transfection cell lines of *GNA13* low-expression (H1299-si*GNA13*) and *GNA13* overexpression (A549-pcDNA-*GNA13*) and thus carried out *in vitro* and *in vivo* functional validation of *GNA13*. We observed that cell proliferation and migration/invasion properties were reduced considerably following *GNA13* knockdown, while overexpression of *GNA13* significantly enhanced these properties *in vitro*. The zebrafish cell-line xenograft model provided valuable insights into tumor growth and metastasis. The involvement of *GNA13* in regulating proliferation and metastasis in lung cancer was confirmed using the zebrafish xenograft model. It can be found that *GNA13* has a greater capacity to regulate the BM of lung cancer cells in zebrafish than it does for cell proliferation and TM. This finding further confirms the correlation between *GNA13* and BM in lung cancer. Although the observed fold change in *GNA13* expression was modest (1.17-fold in M1 vs. M0), it may reflect underlying biological significance through mechanisms such as signal amplification, post-transcriptional regulation, and the influence of cellular subpopulations—features consistent with those of other established oncogenes.

We next sought to mechanistically identify the pathways involved in *GNA13*-mediated lung cancer cell brain metastasis. EMT is the morphological transformation of epithelial cells into a fibroblast or mesenchymal cell phenotype. Many cancer metastases, including lung, gastric, and colorectal cancers, have been reported to be associated with the EMT process (Dian et al., 2021; Feng and Xu, 2021; Nan et al., 2021). Upregulation of GNA13 expression has been linked to gastric cancer (Zhang et al., 2016), colorectal cancer (Pan et al., 2022), renal cell carcinoma (Liu et al., 2017), and pancreatic cancer (Hu et al., 2015) promoting EMT that enhances the proliferation and metastasis of cancer cells. Additionally, studies on the metastatic mechanisms of lung cancer have demonstrated that the JPX/miR-33a-5p/Twist1 axis plays a role in the progression of EMT by activating the Wnt/β-catenin signaling pathway. FOXP3 is a co-activator that enhances the Wnt/β-catenin signaling pathway, promoting EMT, tumor growth, and metastasis in NSCLC (Huang et al., 2019; Pan et al., 2020; Yin et al., 2020; Yang et al., 2017). Therefore, EMT and Wnt/β-catenin signaling pathways are key pathways to target tumor metastasis. However, the relationship between *GNA13* and the Wnt/β-catenin signaling pathway remains unknown. Our results showed that silence of *GNA13* in lung cancer cells led to decreased phosphorylation of Ser9 in GSK3β. The phosphorylation of Ser33, Ser37, and Thr41 in β-catenin, destabilizes β-catenin and triggers its ubiquitylation and degradation. As a result, the expression of β-catenin and active β-catenin (the stable form of the β-catenin protein) decreased. As the concentration of β-catenin in the nucleus decreases, the expression levels of the proteins Snail-1, Snail-2, ZEB-1, and ZEB-2 are also downregulated. This reduction leads to a decrease in the expression of the N-cadherin. In our present study, p-GSK3β, β-catenin, active β-catenin, Snail-1, Snail-2, ZEB-1, ZEB-2, and N-cadherin protein expression were significantly lower in H1299-si*GNA13* cells, while overexpression of *GNA13* resulted in significantly higher levels of these proteins. These results suggest that *GNA13* supports EMT progression via activation of Wnt/β-catenin pathways.

The pathway inhibition or rescue experiments in future studies can provide direct functional validation, which will more robustly establish the causal relationship between GNA13 and the WNT/β-catenin signaling pathway. And it is important to emphasize that these promising results represent an important but preliminary step in understanding *GNA13*’s clinical significance. Rigorous validation through prospective studies that systematically account for potential confounders - including but not limited to tumor staging, patient demographics, treatment regimens, and molecular heterogeneity - will be essential to establish *GNA13*’s independent prognostic value. Future investigations should also explore whether *GNA13*’s predictive power varies across molecular subtypes of lung cancer, and whether it could serve as a therapeutic target for preventing or treating BM.

## 5 Conclusion

In summary, this study reveals that overexpression of *GNA13* promotes the proliferation, migration, and invasion of NSCLC cells. Otherwise, inhibition of *GNA13* inhibits the proliferation, migration, and invasion of NSCLC cells. GNA13 can induce EMT progression in NSCLC by regulating the WNT/β-catenin signaling pathway, thus facilitating the development of BM. These findings indicate that *GNA13* as a novel candidate target for NSCLC BM therapy.

## Data Availability

The datasets presented in this study can be found in SRA database (accession number: PRJNA1332114).
