# Supplementary material for: GNA13 promotes brain metastasis of non-small cell lung cancer and EMT through the WNT/β catenin signaling pathway
Source: Front Cell Dev Biol. 2025 Sep 26;13:1652200. doi: 10.3389/fcell.2025.1652200 (PMC12511054; doi:10.3389/fcell.2025.1652200)
Supplement: Supplementary file 1 [file Table1.docx]

Supplementary Material

**Table S1 Primer Sequences for qPCR.**

| Genes | Forward primer (5′→3′) | Reverse primer (5′→3′) |
| --- | --- | --- |
| *GAPDH* | GAAGACGGGCGGAGAGAAAC | CCATGGTGTCTGAGCGATGT |
| *SCD* | TCTAGCTCCTATACCACCACCA | TCGTCTCCAACTTATCTCCTCC |
| *HMGCS1* | GATGTGGGAATTGTTGCCCTT | ATTGTCTCTGTTCCAACTTCCAG |
| *GNL3L* | ATGCCAATCGAGAGGCTGAAT | TCTCAATGGTCCTGCGTTTTT |
| *SEC61A1* | TCAACGGAGCCCAAAAGTTATT | ACATCCCGGTCATCACATACA |
| *MYC* | TCCCTCCACTCGGAAGGAC | CTGGTGCATTTTCGGTTGTTG |
| *ZNF207* | GGACCAGGAATACCACCTCTG | GTGCTTGAGTCATTGGAGGAAT |
| *NFKBIA* | ACCTGGTGTCACTCCTGTTGA | CTGCTGCTGTATCCGGGTG |
| *AP3S1* | TACCAGCCCTACAGTGAAGATAC | ATCAGTTTGTTGTCAGATCCTCC |
| *ELOV1* | AACTCTTCCGTGCATGTCATAAT | TGCTTTTTCCACCAAAGGTAGG |
| *RPS5* | ATGACCGAGTGGGAGACAG | GCTTTGCGGAAGCGTTTGG |
| *UBQLN1* | AGGACCGAGCTTTGAGCAAC | TGCAGCACTCAGCATTGGT |
| *PSMC5* | AAGGGGCAAGAATGGTGAGG | AGTCGATTTCGTCCATGAAGATG |
| *NAA25* | CCCTTGAACCCACAGATGACA | TTTTGTAACTAACTCCGGTCGG |
| *DYNC2H1* | TCCAACACGATTGAGTTTGGTG | GGTGCGAATACTTGCCGTACT |
| *CAV1* | ACACAGTTTTGACGGCATTTGG | GCAGACAGCAAGCGGTAAA |
| *HIGD1A* | AAGAGGCACCATTCGTACCC | ACCAACAGTCATTGCTCCTACA |
| *COPZ1* | GATGGAGATCGACTTTTTGCCA | TCAGTCCGATGGGTCTTGTTG |
| *TBP* | CCACTCACAGACTCTCACAAC | CTGCGGTACAATCCCAGAACT |
| *CIBAR1* | GCCAAATCTTCGCTGCCTATG | CTGCTTGTCGATAATCCTGAAGT |
| *DNAJA2* | GAGCTGTCCAAAAGTGTAGTGC | GCATCTGTTGTACCATCCCTG |
| *CAV2* | AAGACCTGCCTAATGGTTCTGC | CTCGTACACAATGGAGCAATGAT |
| *PPFIBP1* | ACAAGTGTTCCCGAAGAGTTCC | CACAGTTGGGTATCAACAGTGG |
| *LRRCC1* | GCGGAAGTGGAAAACGAAGAC | CTGATATGCTCTGCAAGCCTTT |
| *SGO2* | AGTTCAGATGTCGATATTGGGGA | CACCCCTTTTGCCATCCAG |
| *PLSCR1* | ATGCTTCTCACCCGGAAACAA | GGTAGCCACTATATCCTGGAGG |
| *TRNT1* | AGTGCTGAACCGTAGGTGGA | TCGGGAGACTGCAACTTCATT |
| *HEATR5B* | AACAGGCAACAGTAATGCGTC | TCTCCACCGCTCTTTAAGAAAC |
| *CHD6* | CTCGGGAAGGCAAGTAAAGC | TGAGAGTGCAGATGTTCGACC |
| *TMEM167A* | TTGCTGCTTATATGTACCTGTGC | GACTCTTCCGTTCACCAATTCT |
| *GMFB* | ATGTTGCCGAAGATTTAGTGGAA | CCACCAGGCGTTTATCCTTGT |
| *PHACTR4* | GAAGCAGACCAGCCCACTAC | CTTGCCAAAGCCTGAGAACTT |
| *RHOA* | GATTGGCGCTTTTGGGTACAT | AGCAGCTCTCGTAGCCATTTC |
| *GNA13* | CCCAAGGAATGGTGGAAACAA | ACCCAGTTGAAATTCTCGACG |
| *PIK3CD* | AGCCGGAAGACTACACGCT | GGTCAGGTGAGGGGTCAAC |
| *PIK3R1* | AAGAAGTTGAACGAGTGGTTGG | GCCCTGTTTACTGCTCTCCC |
| *PIK3R3* | CTTTGCGGAAGGGAGGCAATA | ACCACGGAATTAAATGTCAGAGG |
| *PIK3CA* | CCACGACCATCATCAGGTGAA | CCTCACGGAGGCATTCTAAAGT |
| *PIK3CB* | AGAGCACTTGGTAATCGGAGG | CTTCCCCGGCAGTATGCTTC |
| *HRAS* | GACGTGCCTGTTGGACATC | CTTCACCCGTTTGATCTGCTC |
| *KRAS* | GGACTGGGGAGGGCTTTCT | GCCTGTTTTGTGTCTACTGTTCT |
| *NRAS* | CAGGGAGCAGATTAAGCGAGT | GGGCTTGTTTTGTATCAACTGTC |
| *MAP2K1* | CAATGGCGGTGTGGTGTTC | GATTGCGGGTTTGATCTCCAG |
| *MAP2K2* | AGGTCCTGCACGAATGCAA | CGTCCATGTGTTCCATGCAA |
| *MAPK1* | TCTGGAGCAGTATTACGACCC | CTGGCTGGAATCTAGCAGTCT |
| *RAF1* | GGGAGCTTGGAAGACGATCAG | ACACGGATAGTGTTGCTTGTC |
| *BMPR2* | GACAGGAGACCGTAAACAAGG | CCATATCGACCTCGGCCAATC |
| *SOS1* | AGTGGCATATAAGCAGACCTGG | AAGTGAGTTGTCGAGCAATTTCT |
| *SOS2* | CCGCAGCCTTACGAGTTCTTC | GGATGCACTTGTTCCTGAACC |
